# Telemedicine support aids lower limb reconstruction after combat injury in Ukraine

**DOI:** 10.1093/jscr/rjad403

**Published:** 2023-07-17

**Authors:** Igor P Khomenko, Igor A Lurin, Konstiantyn V Gumeniuk, Gerard McKnight, Vitaliy V Makarov, Volodymyr V Nehoduyko, Eduard Khoroshun, Serhii V Tertyshnyi

**Affiliations:** National Academy of Medical Sciences of Ukraine, Kyiv, Ukraine; National Academy of Medical Sciences of Ukraine, Kyiv, Ukraine; Research and Practical Center of Preventive and Clinical Medicine, State Administrative Department, State Institution of Science, Kyiv, Ukraine; National Academy of Medical Sciences of Ukraine, Kyiv, Ukraine; Medical Forces Command, Ukrainian Armed Forces, Kyiv, Ukraine; Academic Department of Military Surgery and Trauma, Royal Centre for Defence Medicine, Birmingham, United Kingdom; Humanitarian Surgery Initiative, Royal College of Surgeons of England, London, United Kingdom; Kharkiv National Medical University, Kharkiv, Ukraine; Kharkiv National Medical University, Kharkiv, Ukraine; Military Medical Clinical Centre of the Northern Region, Ministry of Defence, Kharkiv, Ukraine; Military Medical Clinical Centre of the Southern Region, Ministry of Defence, Odessa, Ukraine

**Keywords:** trauma, plastic surgery, telemedicine

## Abstract

Since the destructive and illegal full-scale invasion of Ukraine in February 2022, caring for the victims of war trauma has been an essential function of Ukrainian clinicians [ 1, 2]. The authors present a case where using novel dynamic digital thermography (DDT), combined with international telemedicine support, contributed to saving the lower limb of an injured Ukrainian soldier. A male soldier in his 30s presented with a ‘through and through’ fragmentation wound to the right thigh from an artillery shell exploding nearby. After initial haemorrhage control and resuscitation, the patient was transferred to a tertiary hospital. Using telemedicine support, reconstructive surgery was planned and performed successfully using a perforating flap technique. DDT was used pre-operatively to identify a perforating vessel and post-operatively to ensure perfusion of the flap. The patient made a good recovery and was discharged 14 d post-operatively.

## INTRODUCTION

In 2014, the Armed Forces of Ukraine re-organized medical care according to the North Atlantic Treaty Organisation (NATO) standards: [3]

(i) First Level (Role 1) provides immediate first aid and triage.
(ii) Second Level (Role 2) provides resuscitation and damage control surgery.
(iii) Third Level (Role 3) provides specialized medical care including diagnostic and inpatient care.
(iv) Fourth Level (Role 4) provides definitive care; specialist surgical and medical care including reconstruction and rehabilitation.

The Russian invasion has seen an indiscriminate use of high-energy weapons, leading to significant numbers of trauma patients [2]. Severe limb injury is common; with up to 89% of those treated by the National Military Medical Clinical Centre in Kyiv suffering extremity injuries [2]. Significant lower limb defects are challenging cases and obtaining adequate soft tissue coverage is crucial [4].

We present a case where a perforating flap was used to repair a significant soft tissue wound due to a fragmentation injury, and is presented with consent of the patient.

## CASE HISTORY

**Figure 1:**
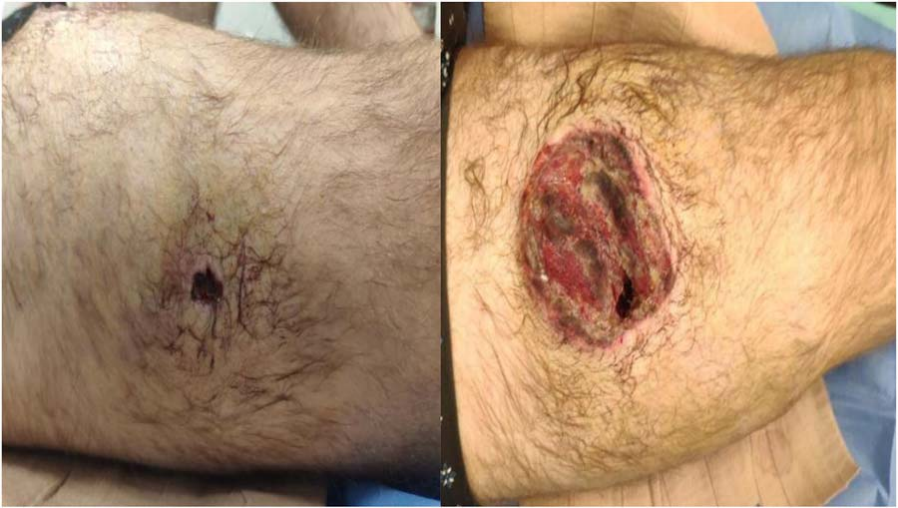
Entry (left) and exit (right) wounds as found at Role 2.

**Figure 2:**
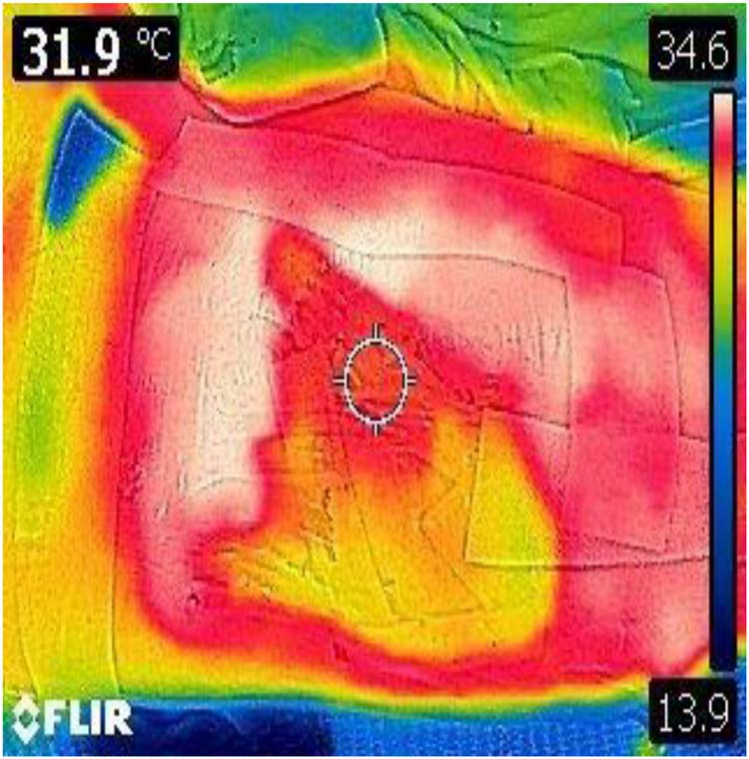
DDT of the wounds surface.

**Figure 3:**
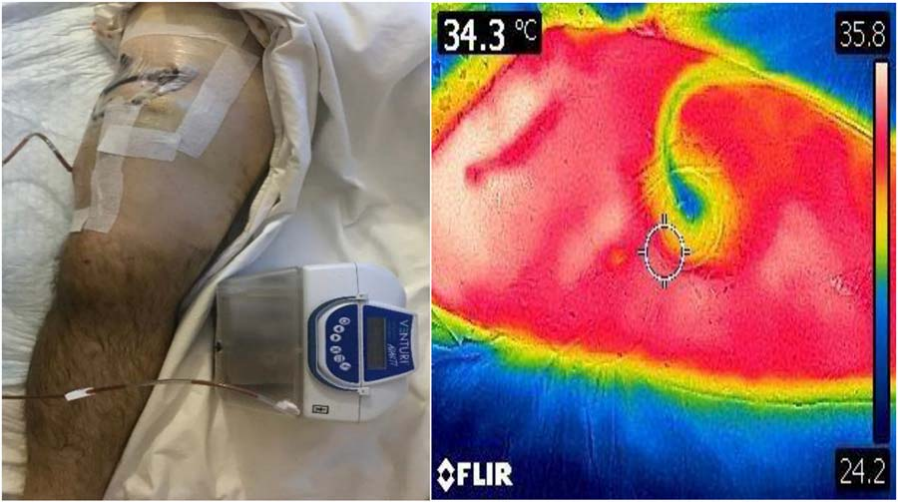
: NPWT in situ with corresponding DDT image.

A 35-year-old male of the Armed Forces of Ukraine suffered a ‘through and through’ fragmentation wound to the right thigh due to an artillery shell in November 2022. At Role 1, he received a Combat Applied Tourniquet and haemostatic dressing. The casualty was evacuated to Role 2 30 min later where examination revealed a 4.5 cm × 1.5 cm entrance wound on the lateral surface of the distal right thigh with a 12 cm × 10 cm × 8 cm exit wound on the anterior surface (Fig. 1). Distal pulses were present and sensation was intact. A radiograph ruled out a fracture and an initial debridement, replacement of haemostatic dressing and limb immobilization was performed under ketamine sedation at Role 2. At day 4, he was evacuated to Role 3 at the Military Medical Centre of the Northern Region of the Armed Forces of Ukraine in Kharkiv. On arrival, the wound was noted be blue-black with necrotic muscle. Grey-yellow granulation tissue was noted in the exit wound that did not bleed; see Fig. 1. A reconstruction plan was devised in conjunction with support from the Charité Clinic in Berlin via videoconferencing. The agreed plan involved repeat debridement and application of a negative pressure wound therapy (NPWT) device, followed by definitive closure with a perforating flap using a branch of the lateral circumflex femoral artery. At debridement, the intra-operative audio Doppler revealed satisfactory blood flow and dynamic digital thermography (DDT) revealed satisfactory temperature of the granulation tissue (Fig. 2) and after pulsed lavage, an NPWT device was applied (Fig. 3).

**Figure 4:**
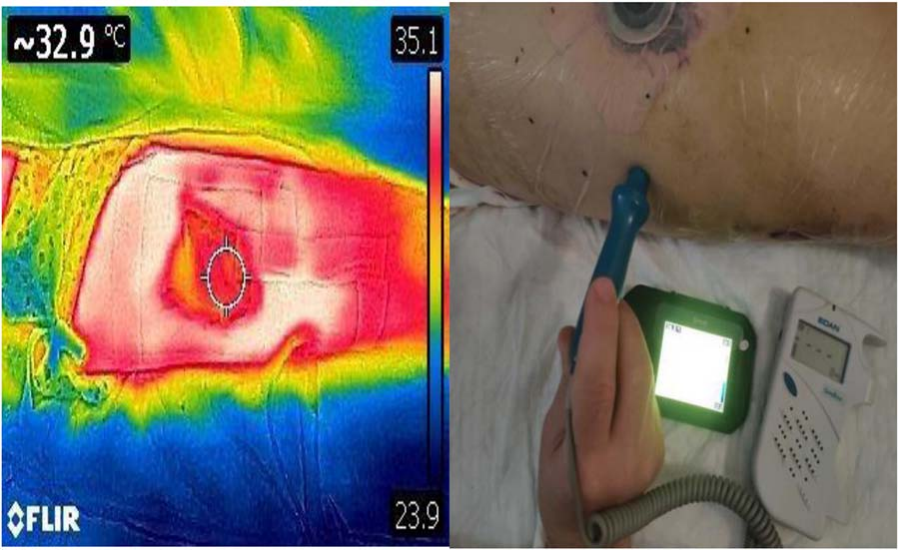
DDT identification of the perforating vessels of the lateral thigh with corresponding confirmation using audio Doppler.

**Figure 5:**
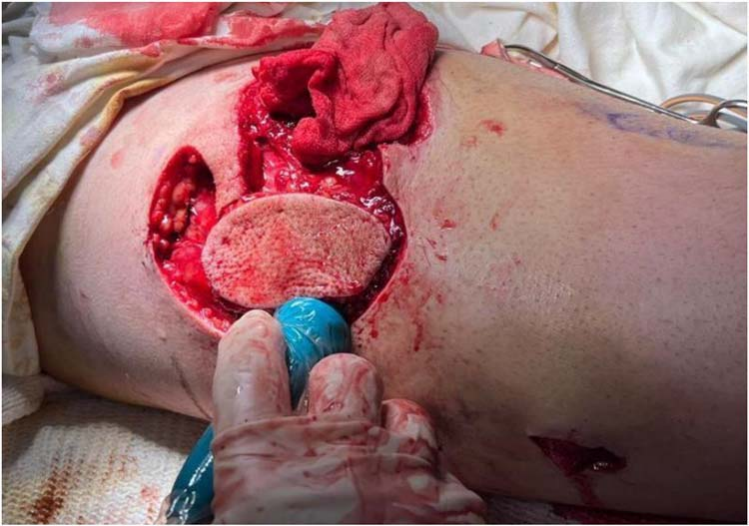
Intraoperative confirmation of the perforating vessel.

**Figure 6:**
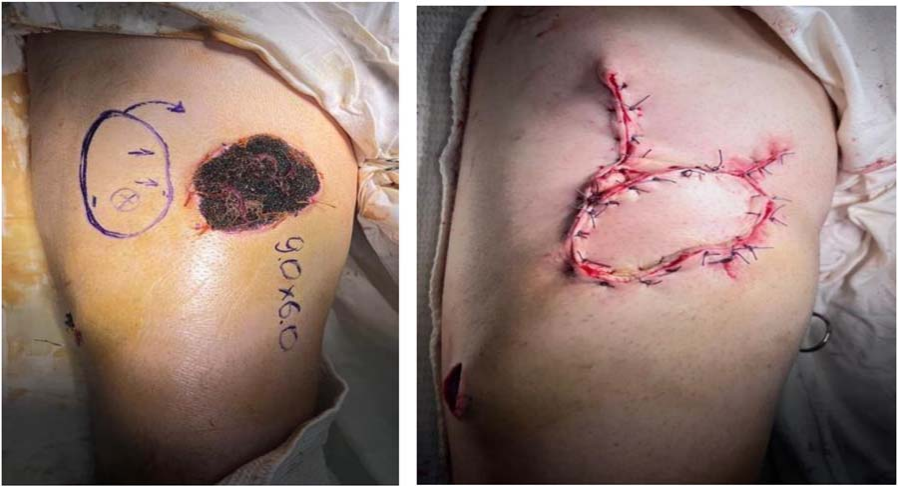
Pre-op marking (left) and immediate post-op appearance (right).

**Figure 7:**
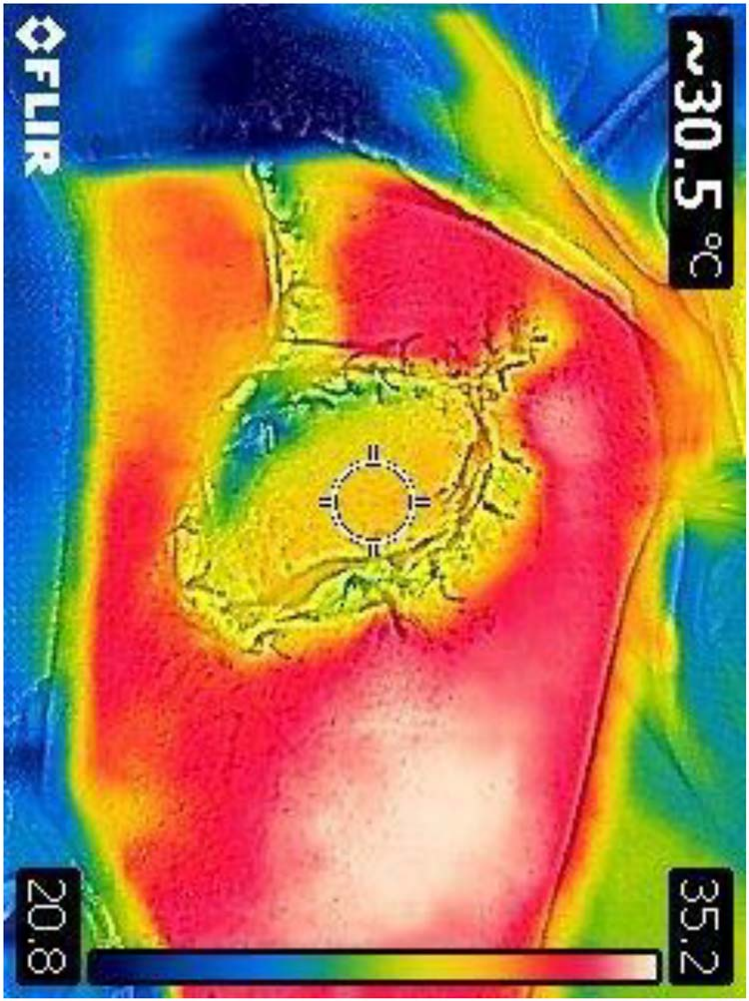
Post-op DDT image confirming perfusion of the flap.

**Figure 8:**
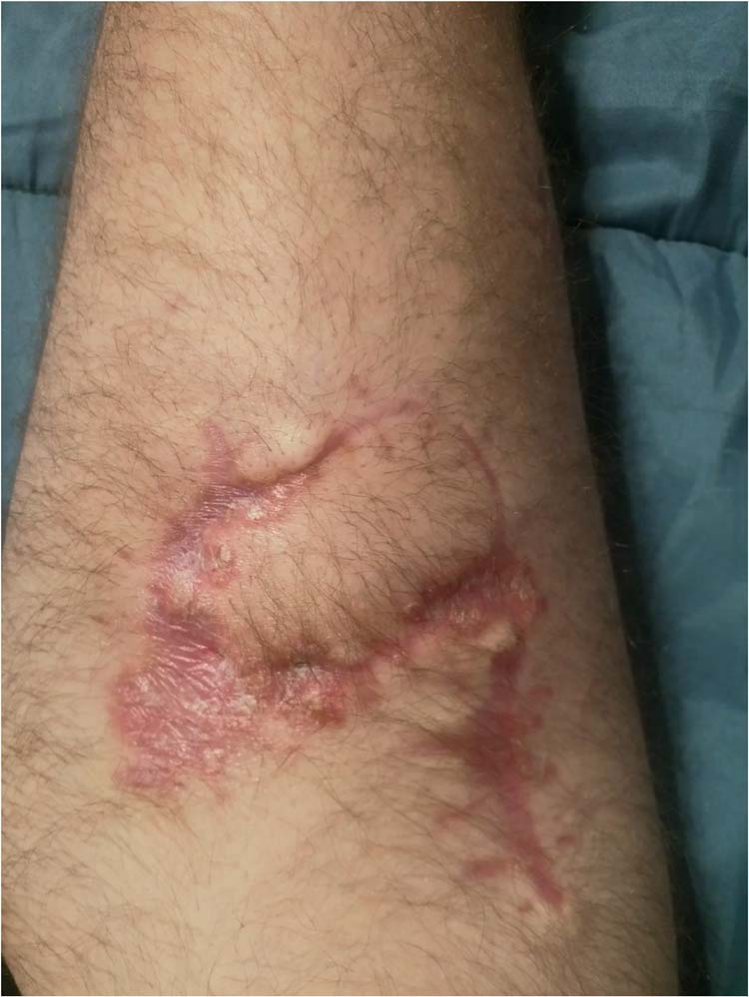
Surgical site at 1 month post-reconstruction.

Prior to reconstruction, a combination of Doppler and DDT was used to locate perforating branches of the lateral circumflex femoral artery (Fig. 4). The flap measured 9 cm × 6 cm × 1.5 cm. A pneumatic tourniquet was used and deflated to allow the Doppler to help select a suitable perforator (Fig. 5). The flap was then raised in the standard technique from proximal to distal, with preservation of the iliotibial band. DDT and Doppler were used at 5, 10 and 15 min to ensure suitable blood supply. The flap was then rotated 90° and sutured to the defect without tension using simple interrupted sutures (Fig. 6). The donor site was closed primarily. DDT and Doppler confirmed satisfactory perfusion at 10 min post-op and an NPWT device was applied. The limb was immobilized, and both DDT and audio Doppler were used every 6 h to monitor the blood supply of the flap for 24 h (Fig. 7). At 14 d post-op, the patient was found to be making excellent progress with no immediate complications. He was allowed to gently mobilize and discharged with guidance to remain on light duties. At 1 month post-op, the wound was healed (Fig. 8) and he returned to active duty in the Armed Forces of Ukraine.

## DISCUSSION

The traditional approach to reconstructing lower limb soft tissue wounds follows the ‘reconstructive ladder’ to improve functional outcomes and reduce the need for amputation [5]. However, the ‘reconstructive elevator’ proposed by Gottlieb and Krieger has been adopted by many colleagues in Ukraine and allows surgeons to choose the optimal method at the first operation [1, 6].

Song et all first described the use of anterolateral thigh flaps in 1984 [7]. Pedicled flaps using the LCFA are widely used due to its versatility and reliability, with overall flap survival rate of 96–97% in ideal conditions in high volume centres [4, 8]. These flaps have been used successfully in Ukraine in austere settings for the closure of military wounds and are favoured as a large donor site is available, there is minimal donor site morbidity, and muscle is preserved leading to good functional outcome [9]. This technique is also efficient, allowing single-stage reconstruction in as little as 45 min [9].

This case demonstrates the utility of combining audio Doppler with handheld DDT. DDT can be used pre-operatively to help identify perforators, and intra- or post-operatively to monitor the flap temperature as a surrogate marker for perfusion. One systematic review concluded that Doppler is better suited to locating deep perforators, whereas DDT is better at locating perforators close to the skin, where its sensitivity and specificity is similar to computed tomography (CT) angiography [10]. In austere military conditions where CT angiography is not readily available, the authors recommend a multimodal approach to planning and monitoring flaps through the use of audio Doppler, DDT and clinical examination.

Finally, the Charité Clinic in Berlin was of great assistance. A number of other international surgical partnerships have been developed in response to the war; statements of support, webinar series, equipment support and deployed surgical care [11–15].

## CONCLUSION

This case has demonstrated that traditional reconstructive surgery techniques can be combined with modern technology and videoconferencing to ensure a good functional outcome. In the authors’ view, DDT can provide a useful adjunct to audio Doppler for patients undergoing flap based reconstructive surgery. DDT is especially useful in the austere military environment where access to CT angiography is severely limited. Additionally, the use of modern teleconferencing can aid decision making and allows for international collaboration.

## References

[1] Sliesarenko S, Badiul P, Sliesarenko D, Korpusenko O. Concept of the lower extremities injuries reconstruction (reconstructive ladder vs. reconstructive elevator). Plast Surg Burn Plast i Oparzenia 2017;5:5–7.

[2] Kazmirchuk A, Yarmoliuk Y, Lurin I, Gybalo R, Burianov O, Derkach S, et al. Ukraine’s experience with management of combat casualties using NATO’s four-tier “changing as needed” healthcare system. World J Surg 2022;46:2858–62. 36070013 10.1007/s00268-022-06718-3

[3] Organisation NAT . NATO Logistics Handbook. NATO Logistics Handbook, Senior NATO Logisticians’ Conference Secretariat, NATO Headquarters, Brussels, 1997. p. 1610–4. Available from: https://www.nato.int/docu/logi-en/1997/lo-1610.htm

[4] Camporro D, García E, Barrio L. Versatility of the lateral circumflex femoral arterial (LCFA) system flaps for lower extremity soft tissue reconstruction. Eur J Plast Surg 2013;36:559–66.

[5] Steinberger Z, Therattil PJ, Levin LS. Orthoplastic approach to lower extremity reconstruction: an update. Clin Plast Surg 2021;48:277–88. 33674049 10.1016/j.cps.2020.12.007

[6] Gottlieb LJ, Krieger LM. From the reconstructive ladder to the reconstructive elevator. Plast Reconstr Surg 1994;93:1503–3. 7661898 10.1097/00006534-199406000-00027

[7] Song YG, Chen GZ, Song YL. The free thigh flap: a new free flap concept based on the septocutaneous artery. Br J Plast Surg 1984;37:149–59. 6713155 10.1016/0007-1226(84)90002-x

[8] Lin C-H, Wei F-C, Lin Y-T, Yeh J-T, Rodriguez EDJ, Chen C-T. Lateral circumflex femoral artery system: warehouse for functional composite free-tissue reconstruction of the lower leg. J Trauma Acute Care Surg 2006;60:1032–6. 10.1097/01.ta.0000218248.22811.70 16688066

[9] Badiul S, Sliesarenko S, Sliesarenko K. The local perforator flaps for plastic closure of extensive military wounds. Plast Surg Burn Plast i Oparzenia 2015;3:59–60.

[10] John HE, Niumsawatt V, Rozen WM, Whitaker IS. Clinical applications of dynamic infrared thermography in plastic surgery: a systematic review. Gland Surg 2016;5:122. 27047781 10.3978/j.issn.2227-684X.2015.11.07 PMC4791361

[11] UK-MED . Our work in Ukraine - UK-MED [Internet]. November 2019 [cited 2023 Jan 2]. Available from: https://www.uk-med.org/ukraine/

[12] England TRC of S of. Humanitarian surgery: Ukraine — Royal College of Surgeons [Internet]. [cited 2023 Jan 2]. Available from: https://www.rcseng.ac.uk/about-the-rcs/international-affairs/humanitarian-surgery-initiative/ukraine/

[13] Association BO . Support for Ukraine - lower limb webinar series [Internet]. [cited 2023 Jan 2]. Available from: https://www.boa.ac.uk/resources/support-for-ukraine-lower-limb-webinar-series.html

[14] ICRC . Ukraine | About the ICRC’s actions in the country [Internet]. [cited 2023 Jan 2]. Available from: https://www.icrc.org/en/where-we-work/europe-central-asia/ukraine

[15] MSF . Ukraine| MSF medical and humanitarian aid [Internet]. MSF. 2021 [cited 2023 Jan 2]. Available from: https://www.msf.org/ukraine

